# Rutaecarpine targets hERG channels and participates in regulating electrophysiological properties leading to ventricular arrhythmia

**DOI:** 10.1111/jcmm.16292

**Published:** 2021-05-03

**Authors:** Ge Zhan, Fang Wang, Yun‐qi Ding, Xiang‐hua Li, Yue‐xin Li, Zheng‐rong Zhao, Jia‐xin Li, Yan Liu, Xin Zhao, Cai‐chuan Yan, Bao‐xin Li

**Affiliations:** ^1^ Department of Pharmacology (State‐Province Key Laboratories of Biomedicine‐Pharmaceutics of China, Key Laboratory of Cardiovascular Medicine Research, Ministry of Education) College of Pharmacy Harbin Medical University Harbin China

**Keywords:** hERG, long‐QT syndrome, phosphorylation, Rutaecarpine, ventricular arrhythmias

## Abstract

Drug‐mediated or medical condition‐mediated disruption of hERG function accounts for the main cause of acquired long‐QT syndrome (acLQTs), which predisposes affected individuals to ventricular arrhythmias (VA) and sudden death. Many Chinese herbal medicines, especially alkaloids, have risks of arrhythmia in clinical application. The characterized mechanisms behind this adverse effect are frequently associated with inhibition of cardiac hERG channels. The present study aimed to assess the potent effect of Rutaecarpine (Rut) on hERG channels. hERG‐HEK293 cell was applied for evaluating the effect of Rut on hERG channels and the underlying mechanism. hERG current (I_hERG_) was measured by patch‐clamp technique. Protein levels were analysed by Western blot, and the phosphorylation of Sp1 was determined by immunoprecipitation. Optical mapping and programmed electrical stimulation were used to evaluate cardiac electrophysiological activities, such as APD, QT/QTc, occurrence of arrhythmia, phase singularities (PSs), and dominant frequency (DF). Our results demonstrated that Rut reduced the I_hERG_ by binding to F656 and Y652 amino acid residues of hERG channel instantaneously, subsequently accelerating the channel inactivation, and being trapped in the channel. The level of hERG channels was reduced by incubating with Rut for 24 hours, and Sp1 in nucleus was inhibited simultaneously. Mechanismly, Rut reduced threonine (Thr)/ tyrosine (Tyr) phosphorylation of Sp1 through PI3K/Akt pathway to regulate hERG channels expression. Cell‐based model unables to fully reveal the pathological process of arrhythmia. In vivo study, we found that Rut prolonged QT/QTc intervals and increased induction rate of ventricular fibrillation (VF) in guinea pig heart after being dosed Rut for 2 weeks. The critical reasons led to increased incidence of arrhythmias eventually were prolonged APD_90_ and APD_50_ and the increase of DF, numbers of PSs, incidence of early after‐depolarizations (EADs). Collectively, the results of this study suggest that Rut could reduce the I_hERG_ by binding to hERG channels through F656 and Y652 instantaneously. While, the PI3K/Akt/Sp1 axis may play an essential role in the regulation of hERG channels, from the perspective of the long‐term effects of Rut (incubating for 24 hours). Importantly, the changes of electrophysiological properties by Rut were the main cause of VA.

## 1 INTRODUCTION

Long‐QT syndrome is characterized by abnormal prolongation of the QT interval which measured by electrocardiogram (ECG) and largely increasing the risk of VF.^1^ LQT can be obtained when drug‐induced delays in cardiac repolarization or other physiological triggers negatively impacting cardiac repolarization, or it can be inherited through gene mutations, resulting in abnormal function of proteins that facilitate cardiac repolarization.^2^ As repolarization of action potential (AP) begins, IKr channels recover from inactivation to ensure the relatively rapid and robust repolarization.^3^ hERG (human ether‐à‐go‐go‐related gene) product was found to be an 1159 amino acid functional K^+^ channel with properties resembling those of IKr.^4^, ^5^ The small central cavity contains extended pockets that can explain its sensitivity to multiple drugs in hERG channels.^6^ Many drugs can block at different sites of hERG channels, which makes it important to analyse the blocking process. From the perspective of different binding sites, Phe656 and Tyr652 are two common sites where drugs bind to hERG channels.^7^ For example, dofetilide suppress I_hERG_ through docking to Phe656.^8^

In recent years, there are many cases of alkaloid drugs causing cardiotoxicity. Aconitine, a major bioactive ingredient in aconite plants is known for its high cardiotoxicity due to the risk of life‐threatening VA.^9^ Many alkaloid drugs have cardiotoxicity risks. Rut is a quinazolinocarboline alkaloid, isolated from Wu‐Chu‐Yu (a dried and unripe fruit of Evodia rutaecarpa), a well‐known Chinese herbal medicine.^10^ Wu‐Chu‐Yu‐based medical products have been widely used in China for hundreds of years to treat gastrointestinal disorders, dysmenorrhea and hypertension.^11^ The reported biological effects of Rut include uterotonic action,^12^ improvement in cerebral functions,^13^ vasorelaxation,^14^ antinociception^15^ and attenuate ventricular remodelling.^16^ The latest research reports that Rut improves the apoptosis and inflammation of peritoneal resident macrophages induced by sepsis.^17^ Nitrogen is located in chemical structure of Rut.^18^ Compared to using different blocking compounds, compounds with structures of nitrogen, hydroxyl or right‐angle are more likely to inhibit hERG channels.^14^ It is generally believed that the main cause of hERG channel obstruction is the hydrophobicity and aromaticity of Tyr652 and Phe656.^19^ Therefore, we speculate that Rut has the potential to inhibit hERG channels.

The members of the Sp1 transcription factor family can act as both negative and positive regulators in the process of gene expression.^20^ It has been reported that Sp1 plays an important role in the transcription of the hERG gene.^21^ Previous studies have shown a great existential discrepancy in the transcriptional regulatory activity of Sp transcription factors family, which is largely determined by the post‐translational modifications including phosphorylation/dephosphorylation.^22^, ^23^ Based on the fact that PI3K/Akt is the upstream of Sp1, which associated with the phosphorylation level of Sp1,^24^ we focused on the PI3K/Akt axis in the present study.

It is not clear whether the extract of evodia rutaecarpa (Rut) has an inhibitory effect on the hERG channels, prolongs the QT interval and eventually leads to arrhythmia. Therefore, it is necessary to study the affinity between Rut and hERG channels. In the research of this topic, we simultaneously used cell‐based and Guinea pig‐based model to assess the effect and mechanism of Rut‐induced cardiotoxicity and arrhythmia risk.

## 2 METHODS AND MATERIALS

### 2.1 Cell culture and plasmid transfection

Human embryonic kidney 293 (HEK293) cells and hERG‐HEK293 cells kindly were donated by the Montreal Heart Institute. The cells were maintained in Dulbecco's Modified Eagle's Medium (DMEM; Hyclone) supplemented with 10% foetal bovine serum (FBS; Bioind). For hERG‐HEK293 cells, 400 μg/mL gentamycin (G418; Thermo Fisher Scientific) was added to the medium. The hERG cDNA mutations, Y652A (tyrosine to alanine) and F656V (phenylalanine to valine), were kindly provided by the Montreal Heart Institute. In brief, cells were plated in dishes at the required density and were incubated overnight. Approximately 3‐4 hours prior to transfection, the media in the dishes were changed with 4.5 mL DMEM. Then, 250 μL opti‐MEM and 12.5 μL lipofectamine 2000 (Thermo Fisher Scientific) were added to the tube labelled A and 250 μL opti‐MEM and 4 μg cDNA were added to the tube labelled B; then, the solutions in A and B were mixed. After incubation at room temperature for 20 minutes, the mixture was added to cells in the dishes. The cells were cultured at 37°C in a humidified CO_2_ incubator. The transfection took place 48 hours prior to analysis.

### 2.2 RNA Extraction and Real‐Time PCR

Total RNA was extracted from the cells incubated with Rut for 24 hours using Trizol reagent according to the manufacturer's instructions (Invitrogen). The RNA samples were stored using DEPC‐treated water. Total RNA was reverse transcribed into cDNA using a ReverTra Ace qPCR RT kit (Toyobo Co Ltd.). Real‐time PCR was performed on the ABI 7500 fast real‐time PCR system (Applied Biosystems) and SYBR Green I (Applied Biosystems). The program of real‐time PCR reaction was carried out at 95°C for 10 minutes followed by 40 cycles at 95°C for 15 seconds, 60 and 72°C for 30 seconds, respectively. The expressions of mRNAs were shown using the comparative cycle threshold (Ct) method (2^−ΔΔCt^ ).

### 2.3 Immunoblot analysis

Wash the cells with phosphate‐buffered saline (PBS) and scrape the cells in cell lysis buffer (Cell Signaling Technology) supplemented with protease inhibitors (Roche Applied Science). Protein lysates from hERG‐HEK293 cells were separated by 8% SDS‐PAGE then transferred to PVDF membrane, and blocked for 2 hours using 5% non‐fat milk and probed with primary polyclonal antibodies against hERG, actin (Zhongshan Jinqiao), Sp1 (Cell Signaling Technology), Akt (Cell Signaling Technology), PI3K (Cell Signaling Technology) were used 4°C overnight. After incubation with secondary antibodies (Li‐CoR), the membranes were examined for bands using the Odyssey Instrument (Li‐CoR). Odyssey v1.2 software was used for quantifying by measuring band intensity and data acquisition.

### 2.4 Immunoprecipitation (IP)

A total for 400 μg of proteins sample from hERG‐HEK cells were incubated with 2 μg Sp1 rabbit antibody or IgG overnight at 4℃ with gentle rocking. Immune complexes were precipitated by incubation with 50 μL ProteinA/G plus beads for additional 1h followed by brief centrifugation. The immunoprecipitates were washed three times with IP buffer, and then subjected to immunoblot analysis with antibody against p‐Ser, p‐Thr (Cell Signaling Technology), p‐Tyr (Cell Signaling Technology).

### 2.5 Immunofluorescence

HERG‐HEK293 cells were seeded overnight on polylysine‐treated coverslips at an appropriate density. The cells were fixed in 4% paraformaldehyde (Solarbio) at room temperature for 30 minutes and washed three times with PBS for 5 minutes each time. Permeate with 0.4% Triton X‐100 for 1 hour at room temperature, blocked with 10% goat serum (Boster) for 1 hour and incubated overnight with rabbit Sp1 antibody (1:1000) and mouse hERG antibody (1:200) at 4°C. The next day, the cells were incubated with diluted Alexa Fluor 488 (mouse) and 594 (rabbit) in the dark at 37℃ for 1 hour, followed by staining of DAPI (Beyotime) for 10 minutes. Finally, cells were observed under a fluorescence microscope.

### 2.6 Electrophysiological recording

The signals were amplified and digitized with an Axopatch 700B amplifier (Axon Instruments) and Digidata 1440B converter (Axon Instruments) at 37℃. Brown Flamming puller (model P‐97, Sutter Instrument Co) was used to pull Microglass pipettes (World Precision Instruments) to resistance 2‐4 MΩ. PClamp 10.6 (Axon Instruments) was used for electrophysiological recordings and analysis of current and AP. IKr was recorded in hERG‐HEK cells.

The extracellular bath solution for hERG currents recordings in hERG‐HEK cells contained 145 mM NaCl, 3.6 mM KCl, 10 mM HEPES, 1 mM MgCl2, 1.3 mM CaCl2 and 5 mM glucose adjusted to a pH 7.4 with NaOH. The intracellular pipette solution for hERG currents recording contained 100 mM K‐aspartate, 25 mM KCl, 5 mM NaCl, 10 mM HEPES, 1 mM MgCl2, 4 mM MgATP and 10 mM EGTA adjusted to pH 7.25 with KOH.

### 2.7 Electrocardiogram record

Male guinea pigs (250 ± 20 g), purchased from Changsheng Life sciences, were randomly divided into control group and Rut group. The animals were kept under standard animal room conditions (temperature 21 ± 1°C; humidity 55‐60%) and fed a standard diet with adlibitum access to drinking water continuously available for 1 week before experiments. All experiments were performed according to the protocols approved by the Institutional Animal Care and Use Committee of Harbin Medical University. Male Guinea pigs were anaesthetized with pentobarbital sodium after being dosed with Rut (25 mg/kg/d) 2 weeks, and BL‐420S biosignal acquisition and processing system (Techman Soft) were used to record ECGs. QT intervals were measured and analysed with the BL‐420S software.

### 2.8 Programmed electrical stimulation

Programmed intracardiac stimulation was performed to assess VF inducibility. The guinea pig was anaesthetized with pentobarbital sodium and place it on the operating table in a supine position. An eight‐electrode catheter (1.5F, Octapolar EP catheter, SciSense Inc) was inserted through the jugular vein into the right ventricle of guinea pig. Intracardiac pacing was performed through the catheter electrodes using an automated stimulator interfaced with the data acquisition system (GY6000, HeNan HuaNan Medical Science & Technology Ltd.). Inducibility of VF was determined by applying a train of ten consecutive electrical pulses with a coupling interval of 70/80/90/100 mseconds (Stimuli 1), followed by an extra stimuli (Stimuli 2) at coupling intervals of 2 mseconds, respectively. Successful induction of VF was defined as the appearance of rapid non‐sinus rhythm ventricular activations lasting for three beats or more.

### 2.9 Optical mapping

Anaesthetized guinea pig was injected with 100 U of heparin intraperitoneally. The hearts were removed and washed in oxygenated (95% O_2_ /5% CO_2_), Tyrode's solution containing 1.3 mM CaCl_2_. Then, hearts were cannulated via the aorta, retrograde perfused and superfused with Tyrode's solution, and arterial pressure was provided by a peristaltic pump with a flow rate of 6‐8 mL/min. Ventricular pacing was achieved with a PowerLab 26T stimulator (AD Instruments). Hearts were loaded with 0.5 µM blebbistatin (Sigma‐Aldrich) to eliminate motion artefact and stained with 0.1 µM voltage‐sensitive dye RH237 (Invitrogen). After 5 minutes, the dye was excited at 710 nm using monochromatic light‐emitting device. Images were acquired with a MiCAM05 CMOS camera (SciMedia) at 2000 frames seconds^‐1^ with a filter setting of 1 kHz. A pseudo‐ECG (p‐ECG) was obtained with electrodes to determine dominant frequency (DF) of VF. The time for a cardiac excitation to travel a certain distance was measured, and conduction velocity (CV) was calculated by the equation: CV = distance / conduction time.

### 2.10 Isolation of guinea pig ventricular myocytes (GPVMs) and solutions for AP recording

Anaesthetized guinea pig was injected with 100 U of heparin intraperitoneally. The rapidly separated heart was transferred to the Langendorf device. The entire process was flushed and retrogradely perfused with Ca^2+^‐free Tyrode's solution (20 mM taurine, 0.4 mM Na_2_HPO_4_, 3.5 mM MgCl_2_, 5 mM HEPES, 5 mM glucose, 5.4 mM KCl and 135 mM NaCl adjusted to a pH 7.4 using NaOH) containing protease (0.1 mg/mL; Sigma), collagenase (1 mg/mL, Worthington Biochemical Co.) and 1% bovine serum albumin (Sigma). The enzyme perfusion process lasts for 10 minutes, and the left ventricle is quickly separated and digested again with fresh Tyrode solution containing collagenase for 8 minutes. Isolated GPVMs were kept in Kraft‐Bruhe (K‐B) solution (3 mM MgCl_2_, 70 mM KOH, 50 mM L‐glutamate, 20 mM KH_2_PO_4_, 20 mM glucose, 10 mM HEPES, 20 mM taurine, 55 mM KCl and 0.5 mM EGTA adjusted to a pH 7.3 with KOH) at 4°C.

Extracellular bath solution for recording AP of GPVMs contained 145 mM NaCl, 5.4 mM KCl, 10 mM HEPES, 1 mM MgCl_2_, 1.8 mM CaCl_2_, 5 mM glucose adjusted to pH 7.4 with NaOH. The intracellular solution contained 120 mM K‐aspartate, 20 mM KCl, 5 mM NaCl, 2 mM CaCl_2_, 5 mM EGTA, 10 mM HEPES and 5 mM MgATP adjusted to pH 7.25 with KOH.

### 2.11 Statistical analysis

All experiments were repeated independently in three or more times under identical conditions. Data are presented as the mean ± SEM. In vitro data analysis was calculated by one‐way ANOVA test, in vivo data were calculated by using two‐tailed Student's *t* test. Calculations were conducted using GraphPad 7.0 (GraphPad software, Inc). ^*^ *P* < .05 was considered statistically significant.

## 3 RESULTS

### 3.1 Acute inhibition of hERG channels by rut

**FIGURE 1:**
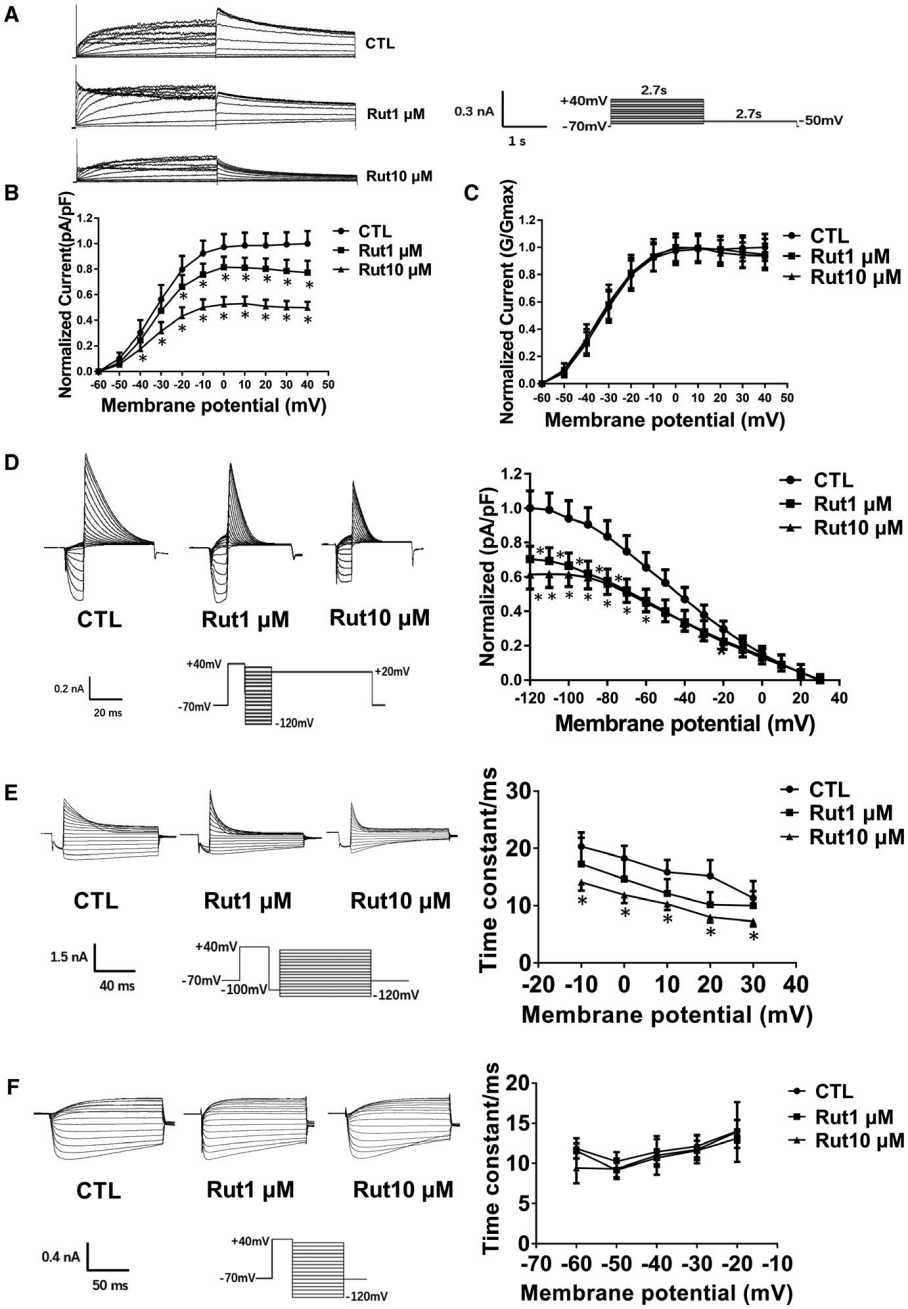
The effect of Rut on hERG channels current and kinetics. A, Protocol and examples on the hERG current under control or Rut‐treated conditions in hERG‐HEK293 cells. Cells are depolarized from a holding potential of −70 mV to voltages in the range −60 to +40 mV and stepped back to −50 mV for 3 seconds to measure the tail currents. B, I‐V curve of the tail current density was the tail current normalized to the capacitance of each cell and maximal current amplitude of CTL group. Rut concentration dependently reduced the hERG current. n = 10. C, Voltage‐dependent activation curves for the control group and group following exposure to 1 μM and 10 μM Rut. Curves were best fits of the data to a Boltzmann function. n = 8. D, Voltage clamp protocol, representative current tracing for steady‐state inactivation (left) and normalized steady‐state inactivation curves (right). Cells are depolarized to +40 mV to ensure all channels are inactivated. The channels are then allowed to relax to a steady‐state of inactivation during a series of short pulses (typically −120 mV to +30 mV). The peak current is then measured at the start of a +20 mV to assess the extent to which channels are inactivated at each voltage of steady‐state. n = 8. E, Voltage clamp protocol, representative current tracing for onset of inactivation (left) and time constant of inactivation curves (right). Cells are depolarized using a pulse of +40 mV to ensure channels are fully inactivated. A short pulse of −100 mV for 25 mseconds is then used to allow channels to recover from inactivation into the open state. The membrane potential is then stepped to a series of test pulses from −120 and +30 mV to test time constant of inactivation at each potential, which can be measured by fitting an exponential function. n = 8. F, Voltage clamp protocol, representative current tracing for recovery (left) and time constant of recovery curves (right). A depolarizing pulse of +40 mV drives all the channels to an inactivated state. The membrane is then repolarized to a series of increasingly negative test pulses from −120 mV to +30 mV. The current is therefore a sum of recovery from inactivation and deactivation which is fit with an exponential to calculate the time constant of recovery. n = 8. Data are presented as means ± SEM. ^∗^ *P* < .05 vs CTL group

In the present study, we attempted to determine the effect of Rut on hERG channels. First, patch‐clamp technique was used to record the I_hERG_ under control condition. Then Rut was washed into the bath system and the current was recorded after 5 minutes (Figure 1). The protocol, examples on the hERG current and current‐voltage (I‐V) curve were shown in Figure 1A,B. Treatment with Rut (1 μM, 10 μM) decreased the tail current amplitude of hERG to 22%, 51% of the control at −50 mV return voltage, respectively. The inhibition of hERG channels by Rut showed a certain concentration dependence. As drugs that inhibit I_hERG_ usually affect the dynamics of hERG channels, we also detected the effect of Rut on hERG channel dynamics. The evidence showed that Rut accelerated inactivation of hERG channels and shorten inactivation time constant (Figure 1D,E). But there was no effect on the activation and recovery process (Figure 1C,F). Taken together, Rut actually inhibited hERG channels, and this effect, at least partially, was attributed to the alterations in hERG channel dynamics

### 3.2 Rut trapped in hERG channels and docked to the amino residues

**FIGURE 2:**
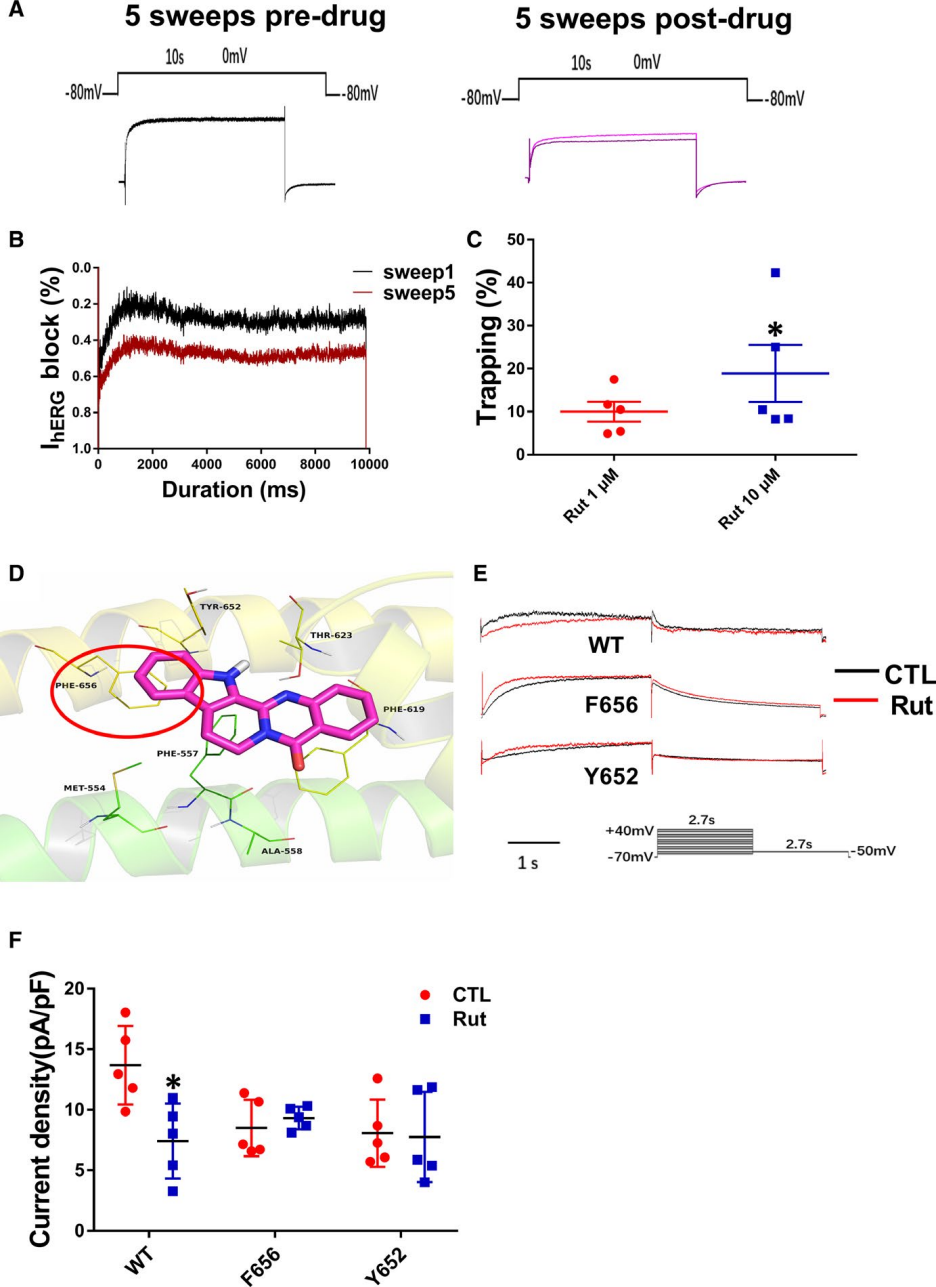
Drug trapped in hERG channels and docked to the amino residues. A, hERG currents were recorded from hERG‐HEK293 cells using whole‐cell patch clamp. Each experiment was divided into three phases: Initially, the 0 mV of 10 seconds voltage pulse was repeated 5 times in the absence of drug to ensure currents were stable and to obtain control current profiles (sweeps 1‐5). Following membrane was holding at −80 mV for 120 seconds without the depolarizing step, while the drug is washed into the system with the channel closed. Finally, a phase of five sweeps of the basic protocol including the depolarizing step to 0 mV was acquired, in the continued presence of drug during which the onset of block is visible as the reduction in the hERG current over the duration of the 10 seconds depolarizing step (sweeps 5‐10). B, Traces represented the first (black) and the last (red) hERG current responded in the presence of 10 μM Rut. C, Statistics of Rut trapped in hERG channels. n = 5. D, The Rut was docked into the binding site of the hERG channels. Autodock vina 1.1.2 was used to simulate binding mode between the Rut and hERG channels. E,F Examples of hERG current traces and statistic of WT‐hERG and mutant hERG current of CTL group and acutely treated Rut groups. n = 5. Data are presented as means ± SEM. ∗ *P* < .05 vs CTL group

Depolarization of the membrane accompanies activation of the hERG channels, but part of the channels could still be bounded by the drug, depending on the degree of trapping that has occurred.^25^ The step depolarization protocol of Milnes et al^26^ was selected as the standard protocol for collecting data representing the extent of drug trapping in the hERG channels. To determine the degree of drug trapping in hERG channels, we evaluated the percentage of channel block at the beginning of the 5th sweep in the presence of the drug. The degree of Rut trapped in hERG channels measured was 10% and 19%, respectively (Figure 2A‐C).

Rut binds to the surface of hERG channel in a tight conformation and is surrounded by amino residues to form a strong hydrophobic bond. Detailed analysis by molecular docking showed that the Rut formed π‐π stacking interactions with the residue Phe‐656 (Figure 2D).

To investigate the binding sites accounting for Rut‐triggered hERG blockage, we tested the effects of the drug on mutant hERG channels (Y652A and F656V). The results showed that Rut has no inhibitory effect on F656V type hERG channels and little inhibitory effect on F652A type hERG channels (Figure 2E,F). These data indicated that interactions with the F656 and Y652 residues were responsible for the blockage of hERG by Rut.

### 3.3 Effects of Rut on the expression of the hERG channels

**FIGURE 3:**
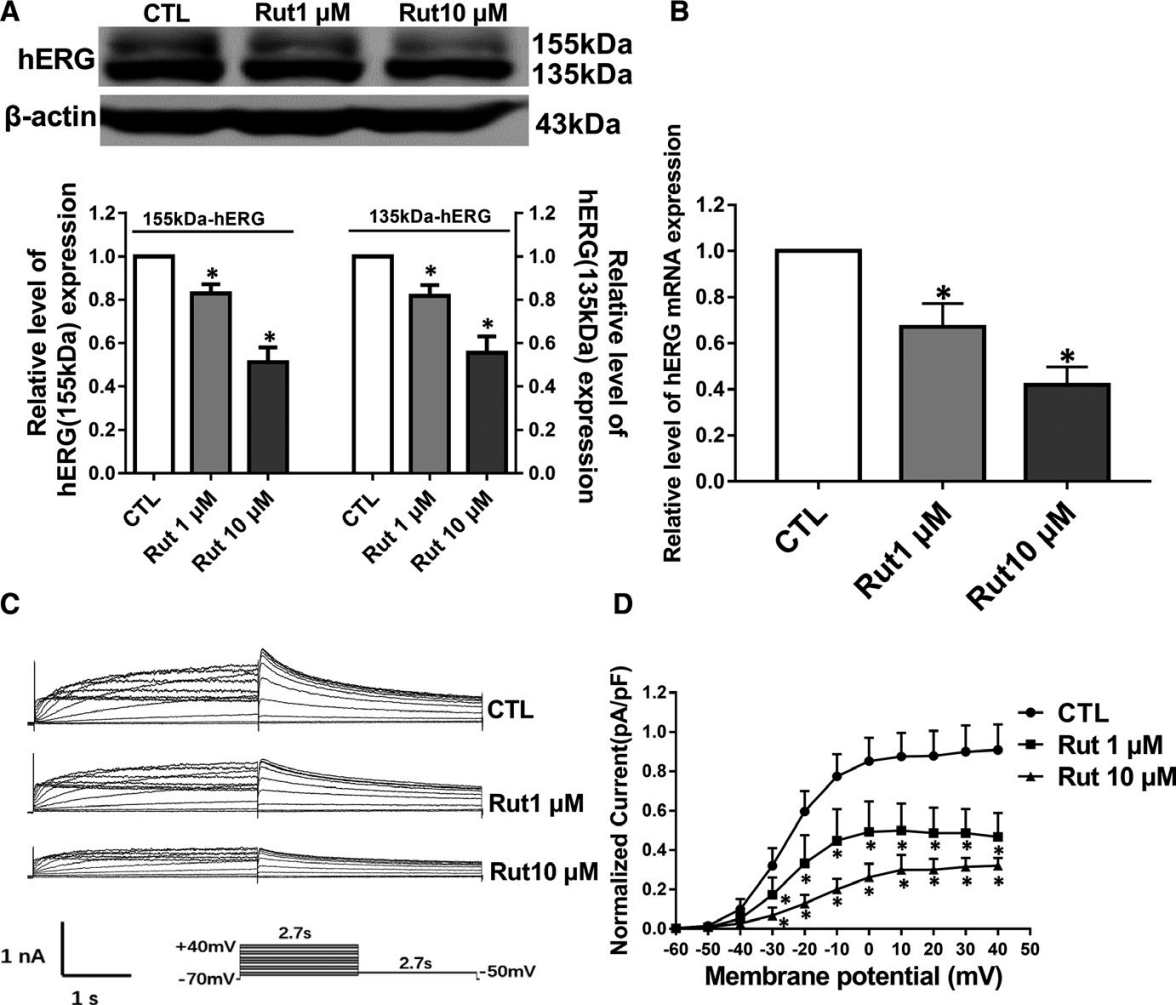
Long‐term effect of Rut on hERG channels. A, Western blot bands and statistic for hERG expression after control or Rut in different concentrations incubation for 24 hours. The expression of hERG channels with molecular weights of 135 and 155 kDa is both suppressed. n = 6. B, Effect of Rut on hERG mRNA levels. Rut reduced hERG mRNA levels. n = 3. C, Protocol and examples on the hERG current under CTL group or Rut‐treated 24 hours in hERG‐HEK293 cells. D, I‐V curve of the tail current density was the tail current normalized to the capacitance of each cell and maximal current amplitude of CTL group. Rut concentration dependently reduced the hERG current. n = 8. Data are presented as means ± SEM. ∗ *P* < .05 vs CTL group

Our current results confirmed that Rut had an acute inhibitory effect on the channels, we next detected whether Rut has a long‐term inhibitory effect on the hERG channels. As shown in Figure 3A, the level of mature 155 kDa hERG and the immature 135 kDa hERG channels decreased after incubation with 1 μM and 10 μM Rut for 24 hours. Simultaneous changes in mRNA expression levels were shown in Figure 3B. Patch‐clamp technique was used to measure the function of drugs on hERG channels. The inhibitory effect of Rut on hERG channels is concentration‐dependent, as shown in Figure 3C,D. The inhibition ratios were 49% (1 μM) and 64% (10 μM) at +40 mV compared with the CTL group, respectively. The results indicated that the suppression of hERG current induced by Rut (incubation for 24 hours) was caused by the decrease of hERG protein expression.

### 3.4 Rut reduced Tyr/Thr phosphorylation of Sp1 through PI3K/Akt and regulated hERG channels expression

**FIGURE 4:**
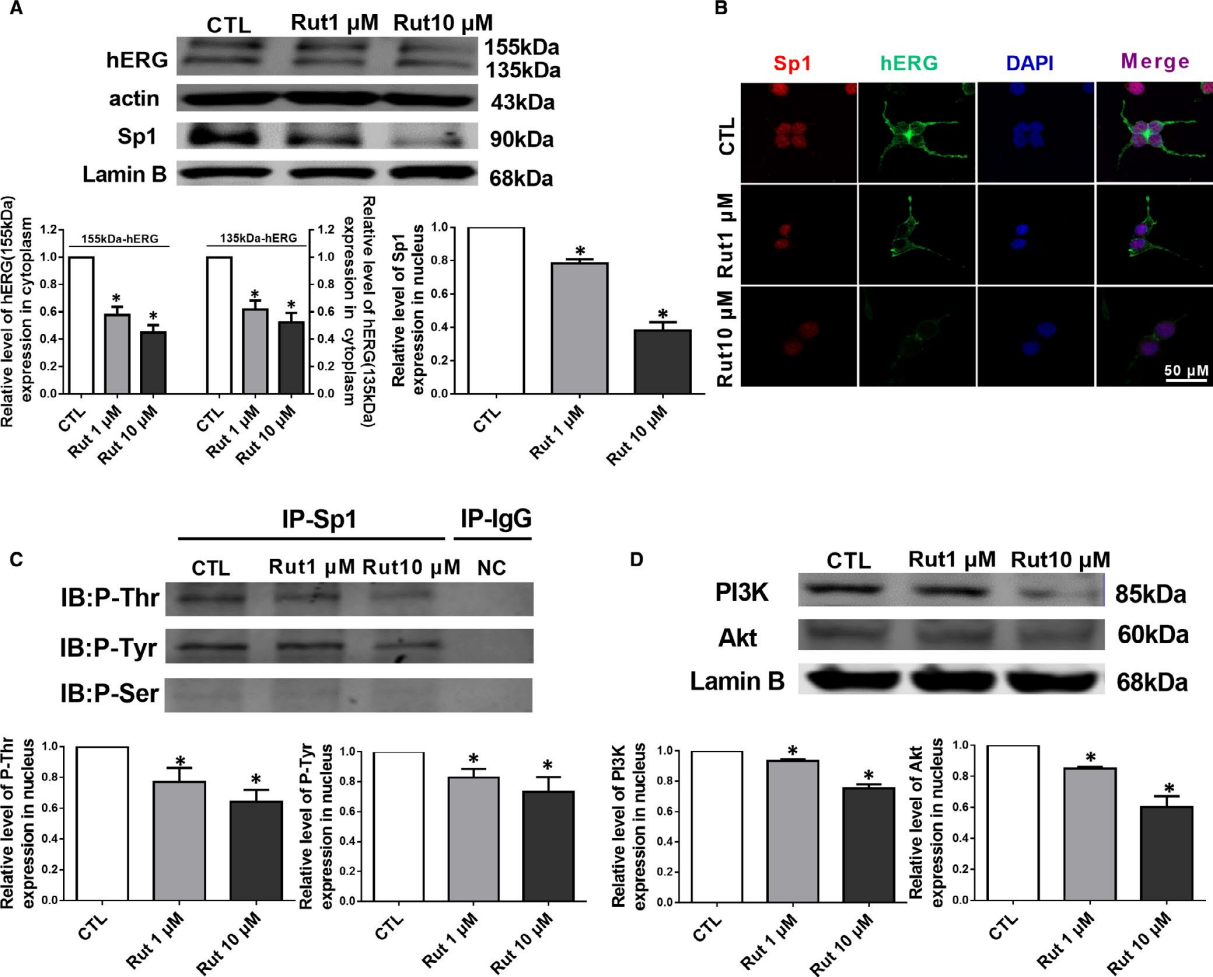
Sp1 phosphorylation is involved in hERG protein down‐regulation induced by Rut. A, Western blot results and statistics for hERG in cytoplasm and transcription factor Sp1 in nucleus expression, Rut reduced the expression of hERG and Sp1 protein in a concentration‐dependent manner (1 and 10 μM). n = 5. B, Immunofluorescence showed reduced hERG protein and Sp1 expression by incubation with Rut. Scale bar, 50 μm. C, Nuclear extracts isolated from untreated and Rut (1 and 10 mM)‐treated hERG‐HEK293 cells were immunoprecipitated with Sp1 or IgG antibody, followed by immunoblotting for phosphorylation. n = 3. D, The effect of Rut on PI3K and Akt in the nucleus of hERG‐HEK293 cells. n = 5. Data are presented as means ± SEM. ∗ *P* < .05 vs CTL group

After separation of nuclear and plasma proteins, it was found that hERG protein in the cytoplasm and Sp1 in the nucleus were reduced (Figure 4A). The transcription factor Sp1 is the downstream of Akt, activated Akt phosphorylates Sp1 to increase its transcriptional activity.^24^ Immunoprecipitation experimental results showed that Thr and Tyr phosphorylation of Sp1 was gradually decreased after treatment with Rut (Figure 4C). However, Ser residue (s) of the Sp1 proteins showed lower level of phosphorylation (No Statistics). To investigate the Sp1 involved mechanism, we evaluated the expression level of Akt and its upstream PI3K. We observed lower expression level of Akt and PI3K after Rut treatment (Figure 4D). Together, these results suggested that phosphorylation of Sp1 through PI3K/Akt signalling pathway was the major mechanism for Rut‐triggered hERG channel dysfunction.

### 3.5 Rut prolonged QT/QTc interval and induced ventricular arrhythmia

**FIGURE 5:**
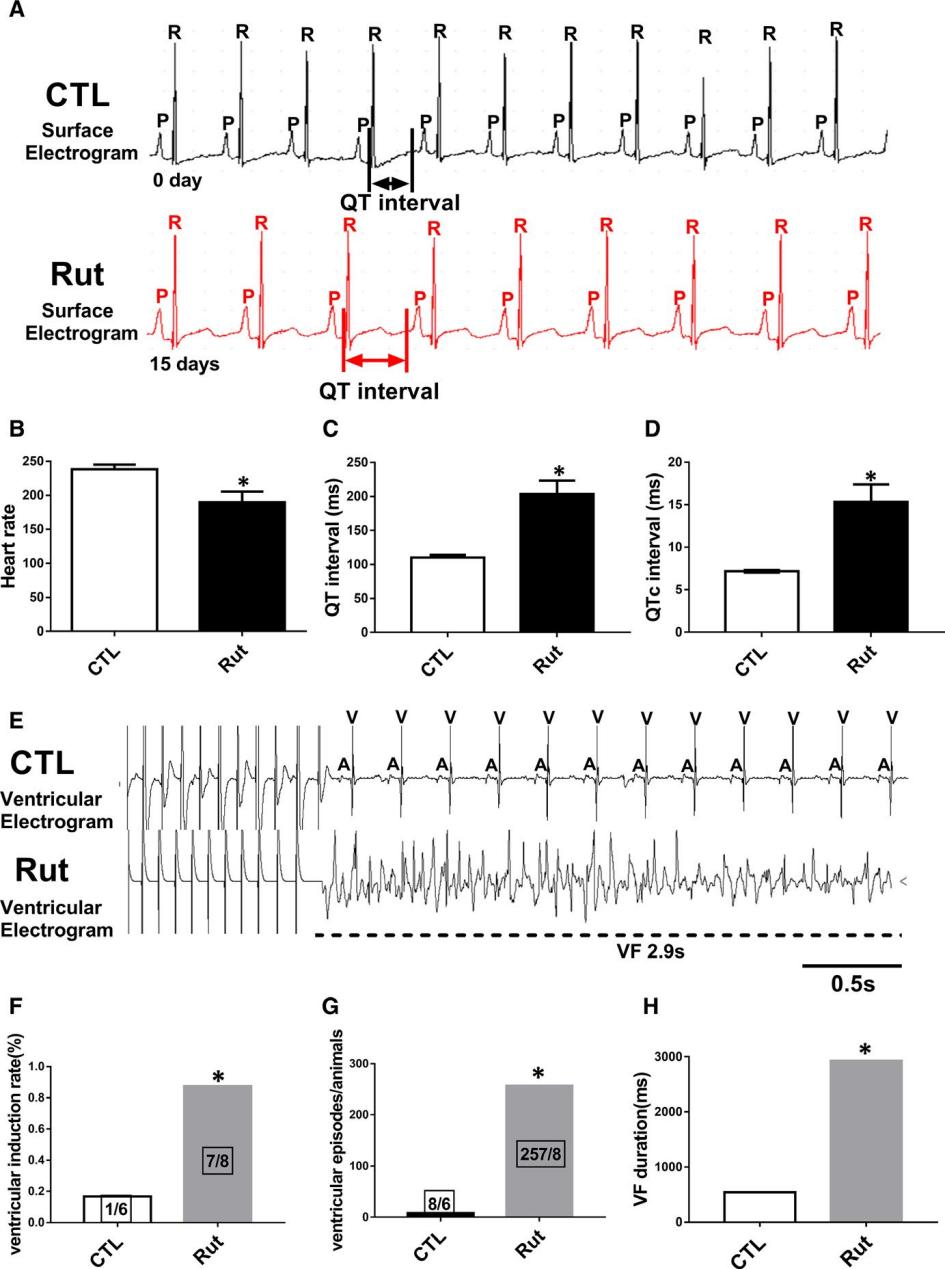
Rut prolonged QT/QTc interval and induced VF. A‐D, Examples and statistics of the ECGs of guinea pigs after being dosed Rut (25 mg/kg) for two weeks. Heart rate was reduced and QT/QTc intervals were prolonged by Rut. n = 6. Data are presented as means ± SEM. E, Representative traces of pacing‐induced VF detected by programmed electrical stimulation. F‐H, Statistics of induction rate of VF, VF episodes and duration of VF. n = 6. ∗ *P* < .05 vs CTL group

To determine whether the inhibition of the hERG channels causes arrhythmia, we performed ECG telemetry recordings. Rut prolonged the QT/QTc intervals of guinea pig compared with the CTL group (Figure 5A‐D).We then evaluated the inducibility of VF in guinea pig dosed for 2 weeks. The induction rate of VF increased from 16.7% in normal group to 87.5% in Rut group (Figure 5F). The VF episodes and duration were also increased in Rut group, which were higher than the CTL group (Figure 5G,H). The duration of VF was up to 2.9 seconds. These data implied that Rut could obviously increase the susceptibility of VF in guinea pig.

### 3.6 Rut decreased conduction and prolong APD in guinea pig heart

**FIGURE 6:**
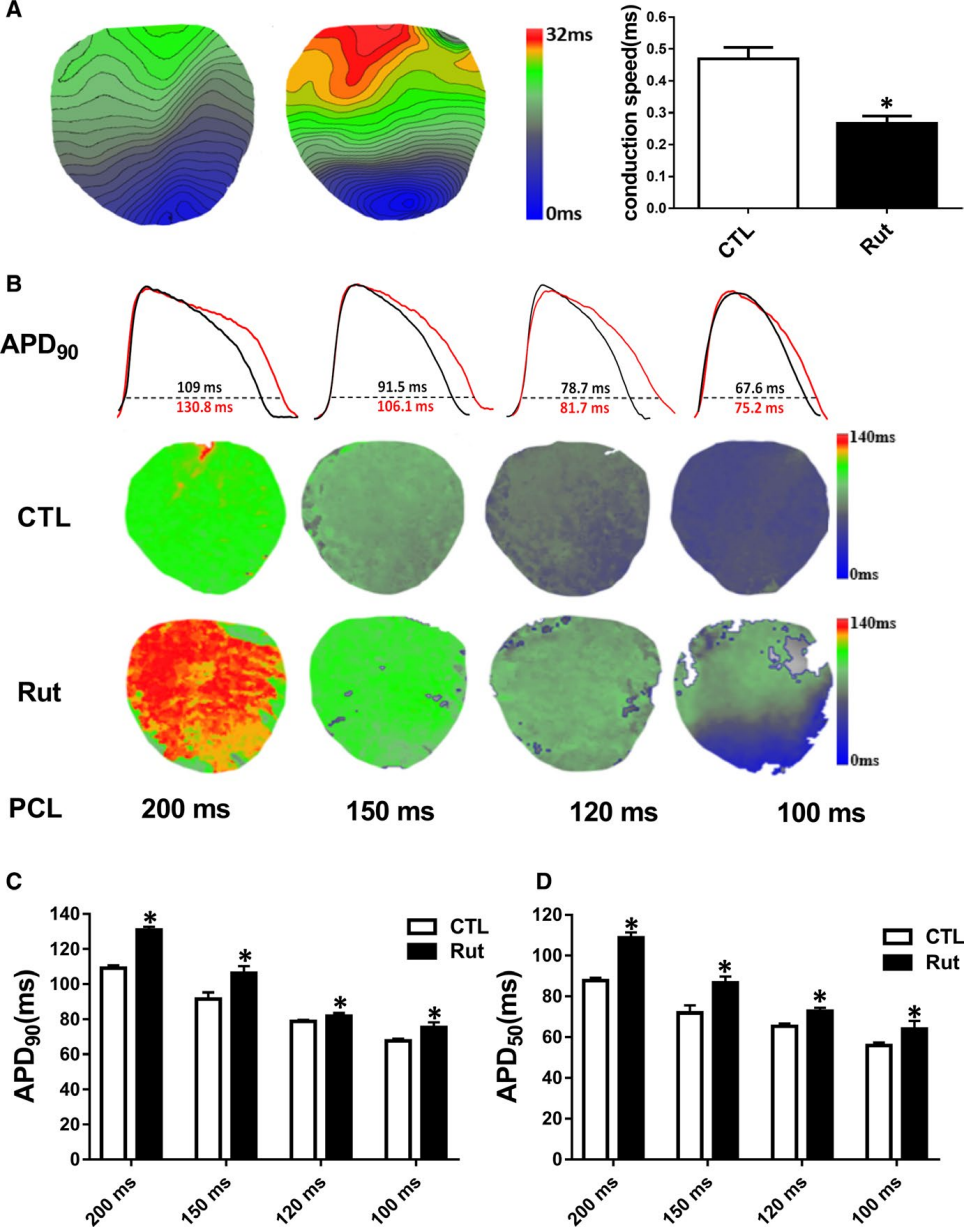
Rut decreased conduction and prolonged APD in guinea pig heart. A, Slowing of CV in guinea pigs after being dosed Rut (25 mg/kg) for two weeks. Optical mapping techniques with a voltage‐sensitive dye to define the cardiac activation. Note that CV was substantially decreased in Rut group. n = 6. B, APD_90_ representative map and lower panel showed Rut prolonged APD_90_ at 200 mseconds, 150 mseconds, 120 mseconds, 100 mseconds PCLs. C,D, Statistical analysis of APD_90_ and APD_50_ of optical mapping. n = 6. Data are presented as means ± SEM. ∗ *P* < .05 vs CTL group

In the present study, Rut increased the incidence of VA, but the mechanism is not yet clear. Thus, we first tried to study the electrophysiological mechanism that caused VA by Rut. To investigate whether Rut could cause electrophysiological disorders, we assessed the effects of Rut on cardiac conduction and APD by Optical mapping. Our results found that Rut slowed down ventricular conduction (Figure 6A). We also evaluated APD by optical mapping, the results showed that Rut prolonged APD_50_ and APD_90_ under electrical stimulation. Specifically, Rut extended the APD_90_ from 190 mseconds, 91.5 mseconds, 78.7 mseconds and 67.6 mseconds to 130.8 mseconds, 106.1 mseconds, 81.7 mseconds, 75.2 mseconds at pacing cycle lengths (PCLs) 200 mseconds, 150 mseconds, 120 mseconds, 100 mseconds, respectively (Figure 6B‐D).

### 3.7 Effect of Rut on VF dynamics

**FIGURE 7:**
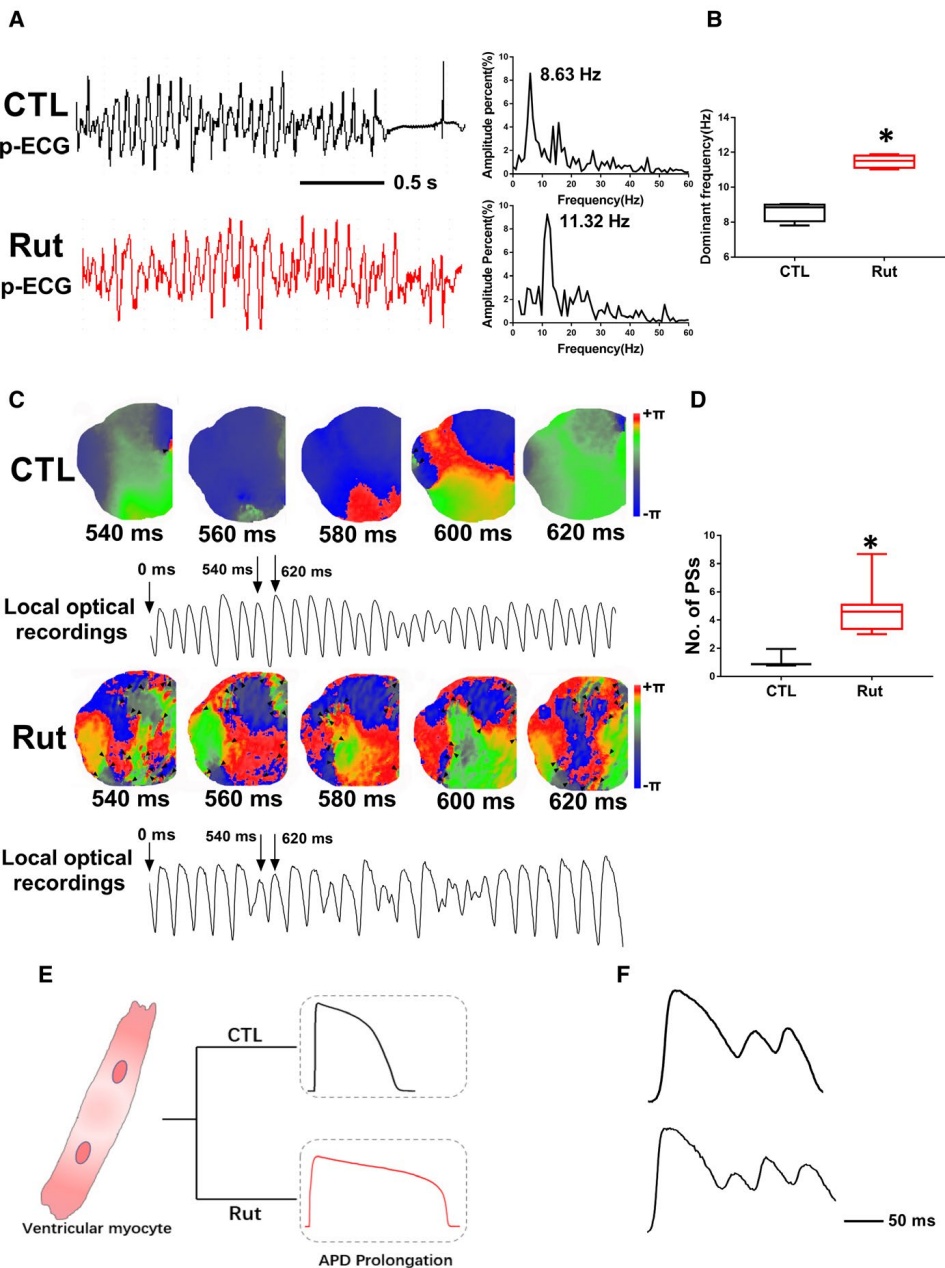
Effects of Rut on DF and PSs of VF. A, p‐ECG recordings of pacing‐induced sustained VF episodes in CTL and Rut groups after being dosed Rut (25 mg/kg) for 2 weeks. Right panel shows the DF distribution of VF. B, DF was increased by Rut. Numbers indicate DF. n = 6. C, Consecutive phase maps sampled at 20 mseconds intervals during the occurrence of VF. PSs are indicated by black arrowheads. Lower panels showed corresponding optical recording of VF. D, Effects of Rut on the number of PSs before and after Rut treatment. The numbers of PSs are increased by Rut. n = 6. E, Representative effects of Rut on APD in isolated single left ventricular myocytes from guinea pig after being dosed Rut (25 mg/kg) for 2 weeks. Statistical was shown in (Figure S2). F, Representative example of EADs in guinea pig hearts after Rut application. Data are presented as means ± SEM. ∗ *P* < .05 vs CTL group

It's well accepted that DF and SPs are dynamic indicators related to VF.^27^ In this study, optical mapping was used to detect the dynamics of VF. Figure 7A showed the p‐ECG recordings of VF in guinea pig heart after being dosed Rut for two weeks. The DF of VF was increased from 8.63 Hz of CTL group to 11.32 Hz (Figure 7B). Consecutive phase maps sampled at 20 mseconds intervals during VF were analysed for PSs (wavebreaks). Figure 7C showed consecutive phase maps with PSs (black arrows), which demonstrated that Rut increased the number of PSs (Figure 7D). Recorded Video (S1A,B) of optical recording showed that the heart electrical activity of the Rut group is abnormal, mainly manifested in irregular activity and messy propagation direction. These data suggested that Rut‐induced VF was mainly manifested in the changes of DF and PSs. Single left ventricular myocyte was isolated for the recording of APD. As shown in Figure 7E, APD was prolonged in the ventricular myocytes from Rut‐treated guinea pigs than normal CTL group. Furthermore, the incidence of EADs was increased by Rut (Figure 7F).

## 4 DISCUSSION

A prolonged QT interval on ECG is flection of delay in ventricular repolarization, which is known as LQTs. LQT predisposes individuals to polymorphic ventricular tachycardia, called torsades de pointes (TdP), which may lead to sudden cardiac death due to VF.^28^ Dysfunction of hERG channels induces LQTs could predispose affected individuals to life‐threatening arrhythmia. Drugs can combine with the ion channels to inhibit the activities of channels.^29^ Researches have confirmed hERG block is one of the most common reasons for commercial withdrawal of drugs.^30^

In our current research, Rut reduced the hERG currents by accelerating the channel inactivation and shorten inactivation time constant (Figure 1). We mutated phenylalanine and tyrosine in hERG channel, which are easy to bind drugs, into non‐aromatic amino acids to observe the potential binding site between drugs and hERG channels. Patch‐clamp results demonstrated that Rut bind to hERG channels through F656 and Y652 instantaneously (Figure 2E,F). Protonized nitrogen forms hydrogen bonds with the oxygen of carbonyl at Tyr623, a half aromatic ring interacts with π–π stacking at Tyr652 and the hydrophobic part can form a hydrophobic interaction with the benzene ring at Phe656, which are the way of drug binding to hERG channels.^7^ Tyr‐652 and Phe‐656, located in the central cavity of hERG channels, are critical for high‐affinity binding of these drugs.^31^

Drug trapping occurs when the drug is not completely dissociated from the channel after repolarization of hERG channels.^32^ Two steps depolarization protocol was chosen as the standard protocol for gathering data describing degree of drug trapping in hERG channels (Figure 2A). The results showed that degree of Rut trapped in hERG channels was 10% and 19%, respectively (Figure 2C). Molecular docking technology (method was shown in supplementary materials) also validated the binding possibility between Rut and hERG channels (Figure 2D). Furthermore, the results of optical mapping showed that Rut reduced APD_90_ and APD_50_ after 10 minutes of acute incubation in guinea pigs hearts, which means Rut has acute effects on AP duration (Figure S3).

In addition to the acute effect of Rut on hERG channel, whether Rut had a long‐term inhibitory effect on hERG channels also needs further research. Interestingly, the present study also showed that Rut could reduce the expression of hERG protein which was a functional protein (Figure 3). Subsequently, we separated the cytoplasm and nuclear protein for analysis. The results of Western blot showed that hERG channels were reduced by incubating with Rut 24 hours and Sp1 in nucleus was inhibited simultaneously (Figure 4A). Immunofluorescence results showed comparable result with Western blot on hERG and Sp1. The results of IP in nucleus demonstrated that Rut reduced Thr and Tyr phosphorylation of Sp1 (Figure 4C) and the expression level of related protein Akt/PI3K was reduced at the same time (Figure 4D). Additionally, intracellular molecular chaperones, like heat shock proteins, participate in the biogenesis of proteins including hERG, such as the process of protein synthesis, folding, assembly and translocation. Previous study has demonstrated that the heat shock protein (Hsp) 70 increases the levels of both immature and mature forms of hERG.^33^ However, Rut did not change the expression level of Hsp70, as well as the interaction between hERG and Hsp70 (Figure S4), indicating that Rut did not interfere with the transport of hERG channels. Collectively, these results suggested that phosphorylation of Sp1 through PI3K/Akt signalling pathway was the major mechanism underlying the Rut‐triggered hERG channel dysfunction in hERG‐HEK293 cell.

The effects of Rut shown above may also imply that Rut had cardiac toxicity and had the potential to cause LQTs or Tdps. To determine if inhibition of the hERG channels could cause arrhythmias, we performed ECG telemetry recordings. The results depicted that Rut slowed down heart rate and prolonged QT/QTc intervals (Figure 5A‐D) in guinea pigs.We then evaluated the inducibility of ventricular arrhythmia by programmed electrical stimulation. The induction rate, episodes and duration of VF were higher than the CTL group (Figure 5E‐H). The duration of VF can last up to 2.9 seconds. These data implied that Rut increased the susceptibility of VA in guinea pig. However, the electrophysiological mechanism of Rut leading to VF still needs to further clarify. As shown in Figure 6B‐D, APD_50_ and APD_90_ in ex vivo guinea pig hearts were prolonged, indicating that Rut had a certain risk of arrhythmia. DF is an important indicator to evaluate the occurrence of arrhythmia.^34^ The DF of each pixel can be represented on a DF map to display different areas with different DF. Phase is a variable that describes the progress of action potential in a specific region of myocardium through a cycle between –π and +π.^35^ The PS displayed on the phase maps was defined as a site with an ambiguous phase surrounded by pixels exhibiting a continuous phase progression from –π to +π. Previous studies suggest that PSs are a reliable alternative representation of wavebreaks, which serve as the source of VF.^27^

In the present study, we found Rut eventually caused VF by increasing the DF (the frequency with the highest power) and the number of PSs (Figure 7A‐D), which mainly affected the dynamics. Furthermore, the incidence of EADs was increased by Rut (Figure 7E). Conventionally, excessively delayed repolarization is linked to arrhythmogenesis in 2 ways. First, at the cellular level excessive ventricular AP prolongation is linked to the development of EADs, depolarizing events arising on the falling phase of the AP. A second way in which delayed repolarization may facilitate TdP arrhythmia is via exacerbation of transmural dispersion of repolarization. A consequence of this is that a given reduction in IKr has a greater effect on midmyocardial repolarization.^4^ In addition, we detected the expression level of hERG, Nav1.5 and Cav1.2. The results in Figure S5 showed that Rut could not change the level of Nav1.5 and Cav1.2, but expression level of hERG in guinea pig heart tissues. These data suggested that Rut‐induced VF was mainly manifested in the changes of DF and PSs and prolonged APD led to increased incidence of EADs.

Collectively, our findings revealed the effect and mechanism of Rut on hERG channels and validated the electrophysiological correlation of cardiac cardiotoxicity in guinea pigs. Our data suggest that we should be more clearly aware of the cardiotoxicity in ventricular arrhythmias caused by drugs, especially by those alkaloid drugs that can target to hERG channels.

## CONFLICT OF INTEREST

The authors declare that they have no conflict of interest.

## AUTHOR CONTRIBUTION

**GE Zhan:** Data curation (lead); Formal analysis (lead); Investigation (lead); Methodology (lead); Software (lead); Writing‐original draft (lead); Writing‐review & editing (lead). **Fang Wang:** Formal analysis (equal); Investigation (equal); Methodology (equal). **Yunqi Ding:** Investigation (equal); Methodology (equal). **Xianghua Li:** Investigation (equal); Methodology (equal). **Yuexin Li:** Formal analysis (supporting). **Zhengrong Zhao:** Investigation (supporting). **Jiaxin Li:** Methodology (supporting). **Yan Liu:** Methodology (supporting). **Xin Zhao:** Methodology (supporting). **Caichuan Yan:** Funding acquisition (lead); Project administration (lead). **Baoxin Li:** Funding acquisition (lead); Project administration (lead); Supervision (lead).

## Supporting information

Supplementary Material

Video S1A

Video S1B

## Data Availability

Data can be accessed by emailing the corresponding authors.
