# Supplementary material for: Rutaecarpine targets hERG channels and participates in regulating electrophysiological properties leading to ventricular arrhythmia
Source: J Cell Mol Med. 2021 May 3;25(11):4938–49. doi: 10.1111/jcmm.16292 (PMC8178274; doi:10.1111/jcmm.16292)
Supplement: Supplementary file 1 — Supplementary Material [file JCMM-25-4938-s002.docx]

**Supplemental materials**

**Expanded Methods**

**Molecular docking**

Molecular docking study was performed to investigate the binding mode between the compound and the human ether-à-go-go-related potassium channel (hERG) using Autodock vina 1.1.2[1]. The three-dimensional (3D) structure of the hERG (PDB ID: 5VA1) was downloaded from RCSB Protein Data Bank (http://www.rcsb.org/pdb/home/home.do). The 2D structure of the compound was drawn by ChemBioDraw Ultra 14.0 and converted to 3D structure by ChemBio3D Ultra 14.0 software. The Auto Dock Tools 1.5.6 package [2,3] was employed to generate the docking input files. The ligand was prepared for docking by merging non-polar hydrogen atoms and defining rotatable bonds. The search grid of the hERG site was identified as center_x: 77.387, center_y: 62.935, and center_z: 81.695 with dimensions size_x: 16, size_y: 16, and size_z: 16. In order to increase the docking accuracy, the value of exhaustiveness was set to 20. For Vina docking, the default parameters were used if it was not mentioned. The best-scoring pose as judged by the Vina docking score was chosen and visually analyzed using PyMoL 1.7.6 software (<http://www.pymol.org/>).

**Statistical analysis**

Data are expressed as mean ± SEM. Comparison between two groups was analyzed by unpaired Student’s t test. Data with more than two groups were analyzed by one-way analysis of variance(ANOVA) .

(**S1 videos are uploaded separately as files**)

**Figure S1 Effect of Rut on electrical activity of guinea pig heart.** (**A**) Video of changes in electrical activity by applying electrical stimulation to induce arrhythmia after administration of guinea pigs for two weeks of CTL group. (**B**) Video of changes in electrical activity by applying electrical stimulation to induce arrhythmia after administration of guinea pigs for two weeks of Rut group.


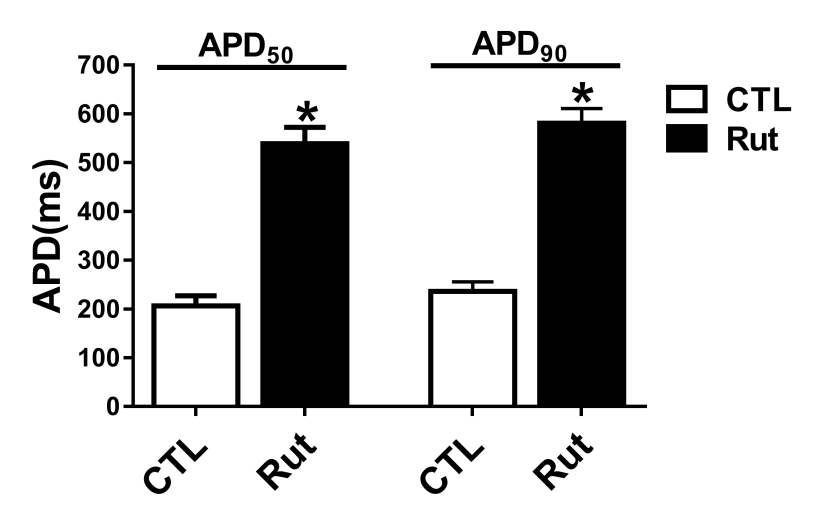


**Figure S2 Rut on APD in isolated single left ventricular myocytes** Patch clamp technique was used to evaluate effect of Rut on APD in isolated single left ventricular myocytes. Statistical showed Rut did prolong APD50 and APD90 in guinea pig after dosing for two weeks. n=6. * P <0.05 vs. CTL group.


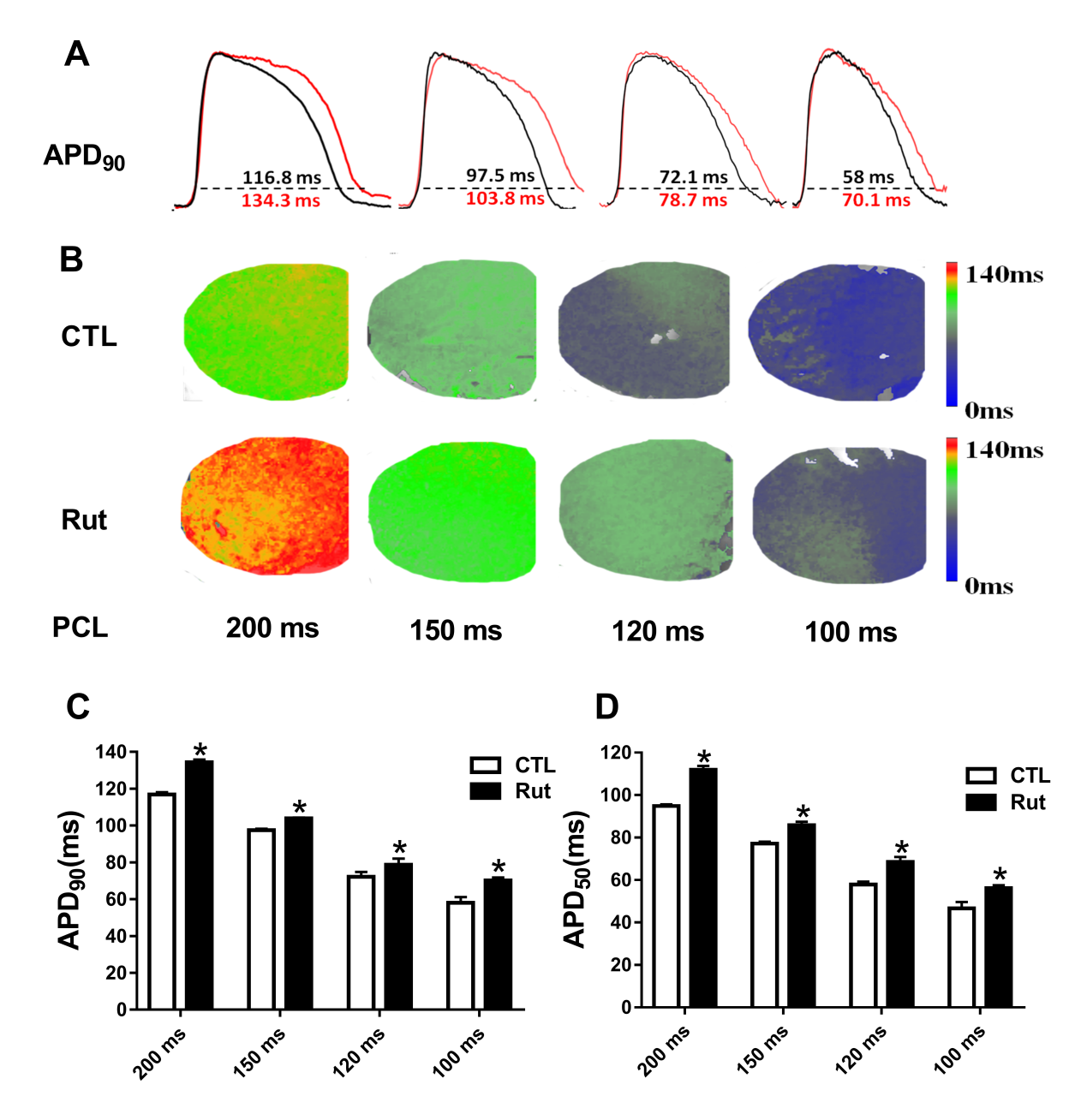


**Figure S3 Acute effect of Rut on APD_90_ and APD_50_ in guinea pig hearts.** (**A-B**) APD_90_ representative maps showed Rut prolonged APD_90_ at 200 ms,150 ms,120 ms,100 ms PCLs. (**C**) Statistical analysis of APD_90_ and APD_50_ of optical mapping. n=6. Data are presented as means ± SEM. ∗ P <0.05 vs. CTL group.

**
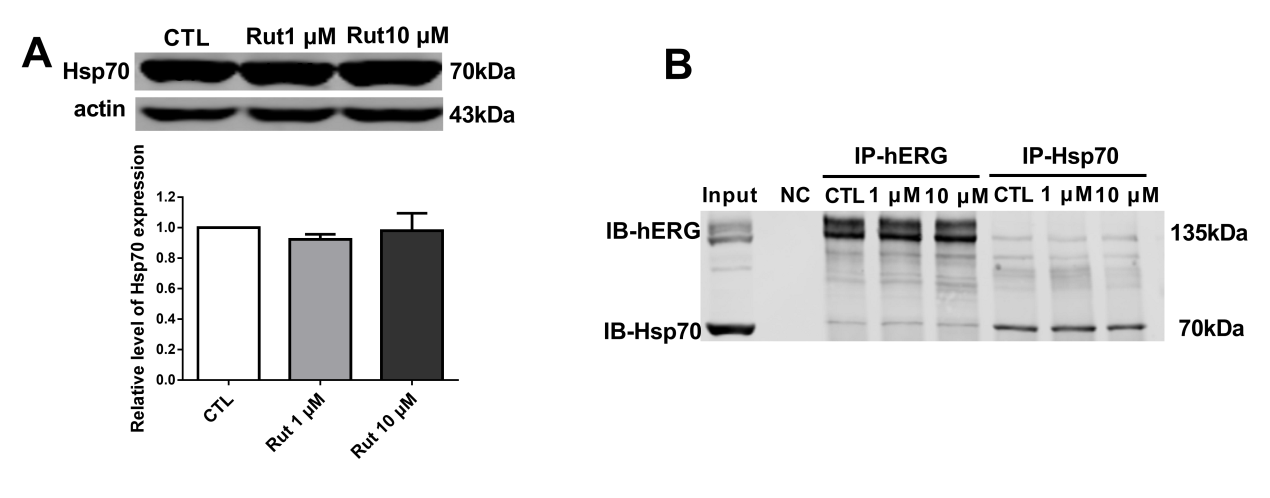
**

**Figure S4 Effect of Rut in hERG channels transport.** (**A**)Western-blot results for Hsp70 expression in the presence of Rut for 24h. The expression of Hsp70 was not changed. n =5. (**B**) Analysis of hERG/Hsp70 complexes formed under control conditions and in the presence of Rut. hERG/Hsp70 complexes were isolated by immunoprecipitation with anti-hERG and anti-Hsp70 antibodies. Rut did not reduce the formation of hERG-Hsp70 complexes.

**
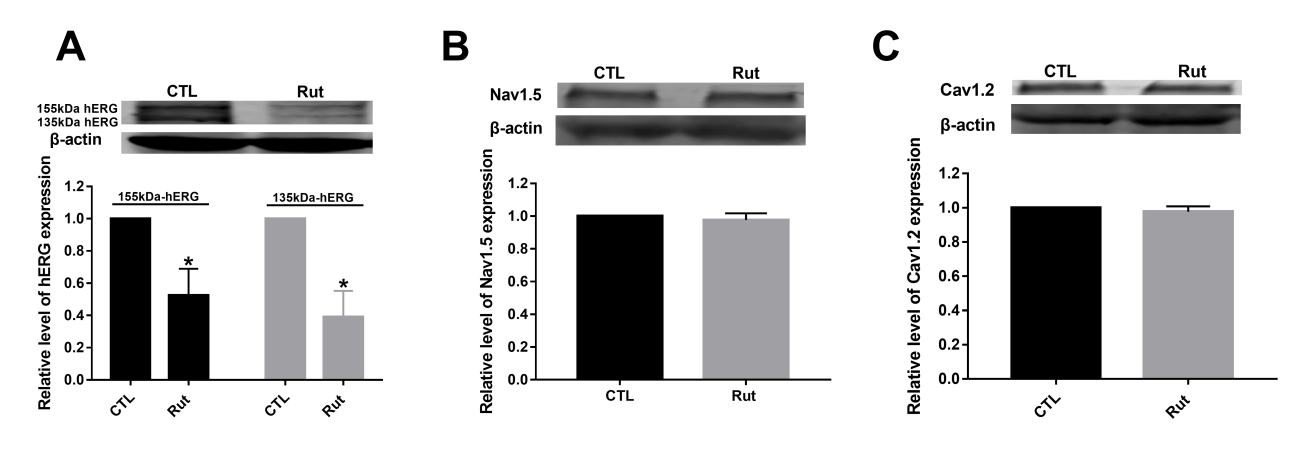
**

**Figure S5 The effect of Rut on ion channels in guinea pig heart.** (**A**) Western blot bands and statistic for hERG expression of control or Rut group in guinea pig heart. n=3. The data are presented as the means ± SEM. *** p<0.05*.* (**B**) Western blot bands and statistic for Nav1.5 expression of control or Rut group in guinea pig heart. n=4. The data are presented as the means ± SEM. * p<0.05*.* (**C**) Western blot bands and statistic for Cav1.2 expression of control or Rut group in guinea pig heart. n=4. The data are presented as the means ± SEM. * p<0.05.

**References:**

1. Trott O, Olson AJ. AutoDock Vina: improving the speed and accuracy of docking with a new scoring function, efficient optimization, and multithreading. *J Comput Chem*. 2010; 31: 455-61.

2. Sanner MF. Python: a programming language for software integration and development. *J Mol Graph Model*. 1999; 17: 57-61.

3. Morris GM, Huey R, Lindstrom W, et al. AutoDock4 and AutoDockTools4: Automated docking with selective receptor flexibility. *J Comput Chem*. 2009; 30: 2785-91.

ssssss
